# Case Report: Congenital Brain Dysplasia, Developmental Delay and Intellectual Disability in a Patient With a 7q35-7q36.3 Deletion

**DOI:** 10.3389/fgene.2021.761003

**Published:** 2021-12-01

**Authors:** Liang-Liang Fan, Yue Sheng, Chen-Yu Wang, Ya-Li Li, Ji-Shi Liu

**Affiliations:** ^1^ Department of Nephrology, The Third Xiangya Hospital of Central South University, Changsha, China; ^2^ Departments of Reproductive Genetics, HeBei General Hospital, ShiJiaZhuang, China; ^3^ Department of Cell Biology, The School of Life Sciences, Central South University, Changsha, China

**Keywords:** 7q terminal deletion syndrome, 7q35-7q363 deletion, SNP array, cerebellar sulcus widening, congenital brain dysplasia, developmental delay

## Abstract

7q terminal deletion syndrome is a rare condition presenting with multiple congenital malformations, including abnormal brain and facial structures, developmental delay, intellectual disability, abnormal limbs, and sacral anomalies. At least 40 OMIM genes located in the 7q34-7q36.3 region act as candidate genes for these phenotypes, of which *SHH*, *EN2*, *KCNH2*, *RHEB*, *HLXB9*, *EZH2*, *MNX1* and *LIMR1* may be the most important. In this study, we discuss the case of a 2.5-year-old male patient with multiple malformations, congenital brain dysplasia, developmental delay, and intellectual disability. A high-resolution genome-wide single nucleotide polymorphism array and real-time polymerase chain reaction were performed to detect genetic lesions. A *de novo* 9.4 Mb deletion in chromosome region 7q35-7q36.3 (chr7:147,493,985–156,774,460) was found. This chromosome region contains 68 genes, some of which are candidate genes for each phenotype. To the best of our knowledge, this is a rare case report of 7q terminal deletion syndrome in a Chinese patient. Our study identifies a rare phenotype in terms of brain structure abnormalities and cerebellar sulcus widening in patients with deletion in 7q35-7q36.3.

## Introduction

The 7q deletion syndrome is a rare genetic disorder caused by the deletion of the long arm of chromosome 7 (Ayub et al., 2016). This 7q deletion was first described in patients with unusual facial structure and delayed mental and physical development, and was consequently defined as a syndrome in 1977 (Harris et al., 1977). The characteristic features of the 7q deletion include developmental delay, intellectual disability, behavioral problems, and distinctive facial features; penoscrotal transposition or ulnar ray deficiency, Kaposi sarcoma, oral malformations, mitral dysplasia, and scoliosis have also been reported (Lewis et al., 1996).

Relatively little is known regarding 7q terminal deletions in contiguous gene deletion syndrome. The typical clinical features of 7q terminal deletion syndrome include abnormal brain and facial structures, developmental delay, intellectual disability, abnormal limbs, and sacral anomalies (Rush et al., 2013; Jackson et al., 2017). At present, 7q terminal deletion syndrome has only been described in 28 patients, most of whom had 7q36 microdeletions (Jackson et al., 2017). This region contains more than 40 OMIM genes, of which *SHH*, *EN2*, *KCNH2*, *RHEB*, *HLXB9*, *EZH2*, *MNX1* and *LIMR1* have been nominated as candidate dosage-sensitive key genes of clinical significance associated with this disorder (Rush et al., 2013; Coutton et al., 2014; Hyohyeon and Lee, 2015; Ayub et al., 2016; Jackson et al., 2017).

Majority of previous published cases were based on traditional G-banding resolution, which is inadequate to define cryptic interstitial deletion in the terminal region. With the development of SNP array technology, which can determine the precise breakpoints instead of terminal deletion, majority of these cases are found as *de novo* in origin. Here, we describe the case of a 2.5-year-old boy with multiple malformations, including congenital brain dysplasia, developmental delay, and intellectual disability, carrying a 9.4 Mb microdeletion in 7q35-7q36.3 (chr7:147,493,985–156,774,460).

### Case Presentation

The patient was a 2.5-year-old boy who first presented to the Department of Pediatrics of Hebei General Hospital due to developmental delay. Both his parents were healthy and were never exposed to undesirable substances, such as poisons and radiation. A family history of birth defects was absent. Pregnancy hypertension occurred at 34 weeks of pregnancy. At that time, B-mode ultrasound suggested that the fetus was 2 weeks less advanced and the head circumference was 3 weeks less advanced than the actual gestational age. Therefore, the mother underwent cesarean delivery.

**FIGURE 1:**
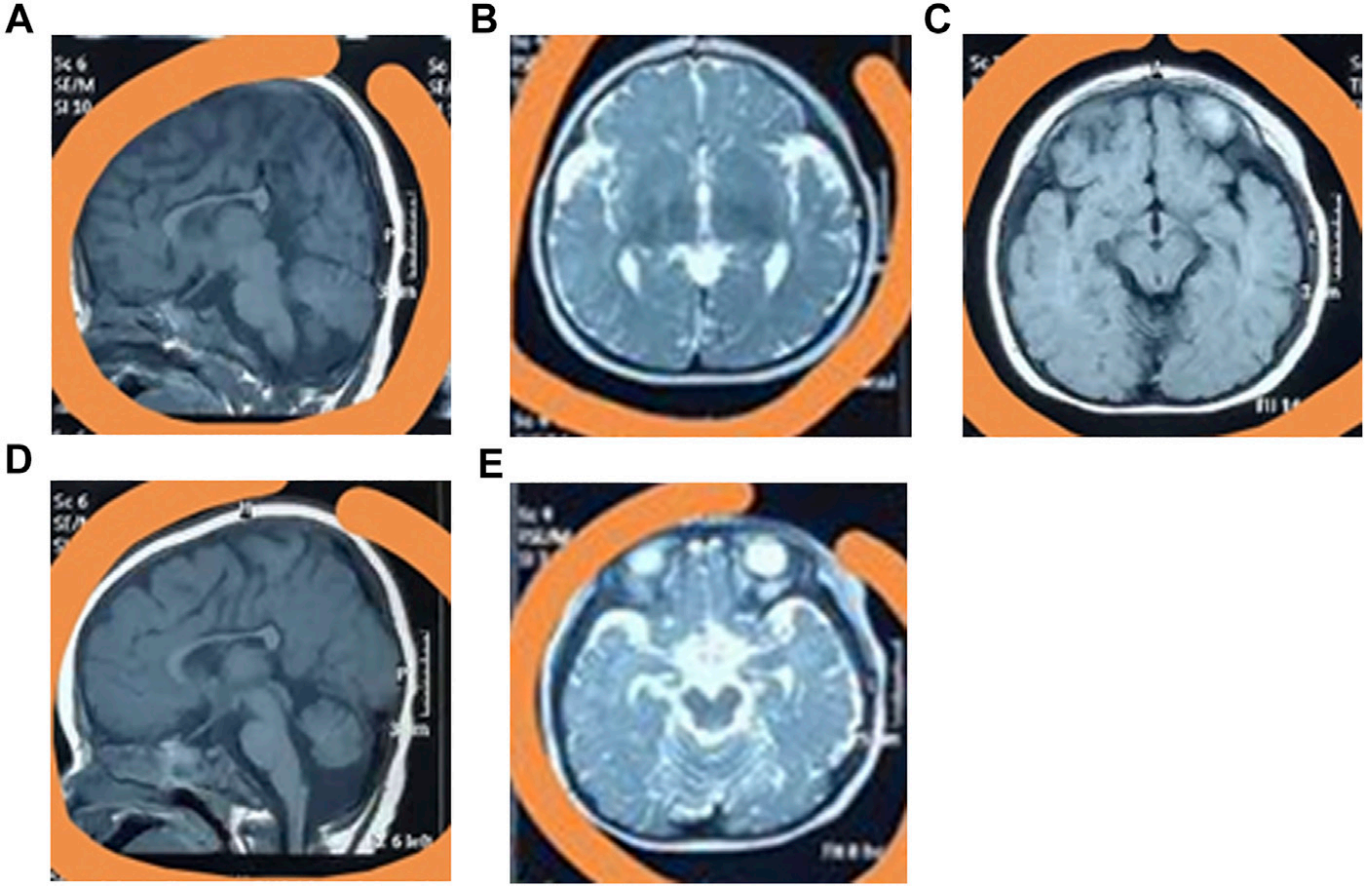
The clinical phenotypes of the patient. The MRI testing identified the overt carcass dysplasia **(A)**, bilateral forehead subarachnoid space widening **(B)**, right iliac choroidal fissure cyst **(C)**, large cisterna magna **(D)**, and cerebellar sulcus widening **(E)**.

The baby was bruised, and exhibited feeding difficulties after birth, with an Apgar score of 6. At six-months-old, he could sit with the help of external objects. At 9 months old, he could not crawl. The baby began to speak at 1.5 years old but could only enunciate simple words, and even now he cannot say full sentences. At present, the patient has a normal weight (11.5 kg) and height (90 cm), but a small head circumference (42 cm), eye crack, broad ears, and a pointed chin (Supplementary Figure S1). Furthermore, the patient cannot walk independently. Brain MRI revealed overt carcass dysplasia (Figure 1A), bilateral forehead subarachnoid space widening (Figure 1B), right iliac choroidal fissure cyst (Figure 1C), large cisterna magna (Figure 1D), and cerebellar sulcus widening (Figure 1E).

**FIGURE 2:**
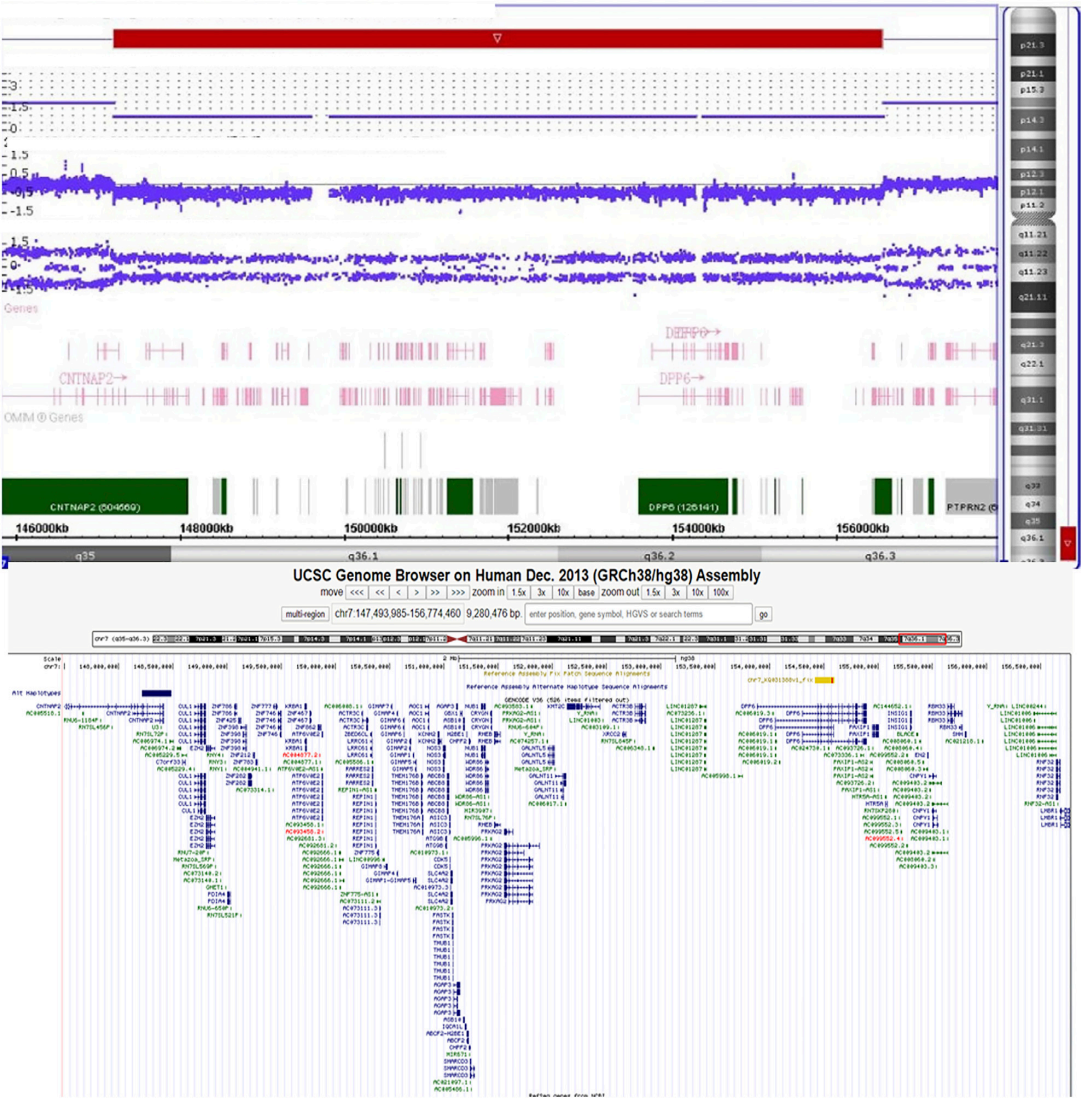
The SNP array identified a 7q35-7q36.3 (chr7:147,493,985–156,774,460) deletion in the patient.

Banding cytogenetic results of the patient revealed a deletion of the long arm of chromosome 7, described as 46,XY,del (7)(q36). His parents’ karyotypes were normal (Supplementary Figure S2). We subsequently performed single nucleotide polymorphism (SNP) array with Human660W-Quad Chip (Illumina Inc., San Diego, United States) to analyze any genetic lesions. A total of 173 CNVs were identified in the proband. Compared with the database of Genomic Variants, a *de novo* 9.4 Mb deletion ranging from 7q35 to q36.3 (chr7:147,493,985–156,774,460) (hg 38) was detected (Figure 2). This chromosome region contains approximately 68 genes, including *CNTNAP2*, *AGAP3*, *CDK5*, *CUL1*, *KMT2C*, *XRCC2*, *DPP6*, *HTR5A*, *EN2, SHH*, *LMBR1*, *KCNH2*, *PRKAG2,* and *EZH2*. The patient’s parents did not carry this genomic lesion. Real-time quantitative polymerase chain reaction with part of the genomic DNA (SHH gene, the primers were as follows: forward: 5-GCA​AGT​GGC​AAC​TCA​CCT​A-3, reverse: 5-TTT​ATT​TAC​CTC​AGG​CCC​TAA​CC-3) of the trio (the proband and his parents) further confirmed this *de novo* deletion (Supplementary Figure S3).

## Discussion

7q terminal deletion syndrome is a rare disorder worldwide. Currently, there have been very few reports in the Chinese population. In this study, we report a heterozygous 9.4 Mb microdeletion of 7q35-q36.3 (chr7:147,493,985–156,774,460) in a 2.5-year-old boy with congenital brain dysplasia, developmental delay, and intellectual disability. The findings of our study are consistent with those of previous studies and report that microdeletion in the 7q terminal may lead to abnormal brain and facial structures, developmental delay, and intellectual disability (Linhares et al., 2014; Busa et al., 2016).

There are several significant genes located in the region of 7q35-q36.3 (chr7:147,493,985–156,774,460). Previous studies have shown that mutations in *CNTNAP2*, *KMT2C*, *EN2* and *EZH2* can lead to intellectual disability and autism spectrum disorder (Penagarikano et al., 2011; Sundaram et al., 2014; Koemans et al., 2017; Suri and Dixit, 2017); *CDK5* is required for proper development of the mammalian central nervous system (Alvarez-Periel et al., 2018), *AGAP3* can regulate synaptic plasticity (Oku and Huganir, 2013), *XRCC2* is required for embryonic neurogenesis (Deans et al., 2000), DPP6 mutations may explain the lateral sclerosis (van Es et al., 2008), and *HTR5A* is a candidate gene for schizophrenia (Guan et al., 2016). These findings may explain congenital brain dysplasia and intellectual disability phenotypes. Furthermore, *DPP6* encodes a dipeptidyl-peptidase-like protein expressed predominantly in the brain, with very high expression in the cerebellum, which may explain the new phenotype of cerebellar sulcus widening (van Es et al., 2008). Finally, *CUL1* can regulate the β-catenin and Wnt pathways, which play a crucial role in body development (Wei et al., 2007).

In fact, another four genes (*SHH*, *LMBR1*, *KCNH2*, and *PRKAG2*) may also affect the phenotypes of 7q terminal deletion syndrome (Hyohyeon and Lee, 2015; Jackson et al., 2017). First, *SHH* and *LMBR1* are responsible for bone and tooth development; therefore, most 7q terminal deletion patients may show microcephaly, abnormal hand, and scoliosis (Rush et al., 2013). In our case, the head circumference was smaller than that in normal individuals, which may have been caused by the haploinsufficiency of the *SHH* and *LMBR1* genes. In addition, two other genes of interest were *KCNH2* and *PRKAG2*; *KCNH2* is a candidate gene of Long QT syndrome (Tuveng et al., 2018) and mutations in *PRKAG2* may lead to hypertrophic cardiomyopathy (Porto et al., 2016). However, most 7q terminal deletion patients show no obvious cardiovascular disorders. In our study, the patient also did not have cardiovascular disease, but we think 7q terminal deletion patients may have a high risk for the future development of cardiovascular disorders, and we will continue to follow the patient.

**FIGURE 3:**
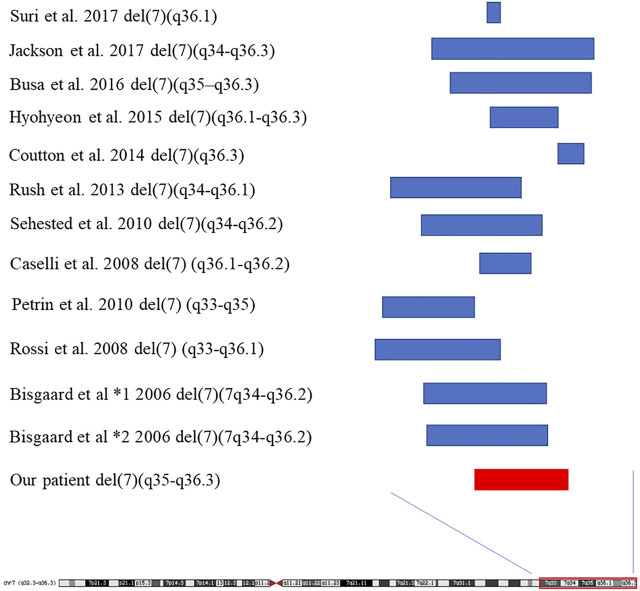
The sumary of reported cases with 7q35-7q36 deletion.

We summarized the 16 reported patients with 7q35-7q36 microdeletion, and found that facial deformities, growth retardation, intellectual disability, speech delay, and poor attention were the common phenotypes in 7q35-7q36 microdeletion patients (Table1; Figure 3), and the 7q36.1-7q36.3 including *EZH2*, *MNX1* and *SHH* may be the critical region of the 7q deletion syndrome which is responsible for facial malformation, developmental delay and intellectual disability (Coutton et al., 2014; Hyohyeon and Lee, 2015; Suri and Dixit, 2017). However, other phenotypes, including abnormal limbs, hearing loss, seizures, short stature, heart defects, and urogenital anomalies have been rarely reported. Meanwhile, most cases with 7q35-7q36 microdeletion have been reported in the United States population (Roessler et al., 1996). Compared to reported cases with 7q35-7q36 microdeletion, we did not observe any limb abnormalities, hearing loss, seizures, short stature, heart defect, or urogenital anomalies in our case. Simultaneously, brain structure abnormalities were only reported in three cases with 7q35-7q36 microdeletions. Caselli et al. reported the hypoplasia of the corpus callosum in a 9-year-old girl with a 5.27 Mb deletion in 7q36.1-q36.2 (Caselli et al., 2008). In 2010, Petrin et al. described cerebellar atrophy in a Brazilian stuttering case with a 10 Mb deletion of chromosome region 7q33-35 (Petrin et al., 2010). Afterwards, Pelegrino et al. reported the hypoplasia of corpus callosum and white matter reduction in a child with a deletion of 7q36.1–36.3 and duplication of 9p22.3–23 (Pelegrino et al., 2013). All the reported 7q35-7q36 microdeletions cases brain structure abnormalities were shown hypoplasia of the corpus callosum. Here, in our study, the case not only presented with hypoplasia of the corpus callosum, but also showed cerebellar sulcus widening, which has not been reported in previous 7q35-7q36 microdeletion patients.

**TABLE 1:** The summary of reported patients with 7q35-7q36 microdeletions.

Patient reported	Our patient	Suri and Dixit. (2017)	Jackson et al. (2017)	Busa et al. (2016)	Hyohyeon and Lee. (2015)	Coutton et al. (2014)	Rush et al. (2013)	Pelegrino et al. (2013)
Sex	M	M	F	M	M	M	F	M
Age	2.5 years	13 years	16 years	2 years	13 years	-	12 years	-
Cytogenetic location	7q35-q36.3	7q36.1	7q34-q36.3	7q35–q36.3	7q36.1-q36.3	7q36	7q34-q36.1	7q36.1-q36.3
Size of deletion	9.4 Mb	1.2 Mb	16 Mb	14 Mb	6.89 Mb	2.7 Mb	13.2 Mb	-
Brain structure abnormalies	carcass dysplasia; bilateral forehead subarachnoid space widening; right iliac choroidal fissure cyst; cerebellar sulcus widening	-	-	-	-	-	-	hypoplasia of corpus callosum; white matter reduction
Facial features	small head circumference; eye crack; broad ears; pointed chin	hypertelorism with downslanting palpebral fissures; coarse hair; full lips	dental malposition	bitemporal narrowing; upslanting palpebral fissures; bulbous nose; down turned corners of the mouth	downslanting palpebral fissures; a bulbous nasal tip	congenital nasal pyriform aperture stenosis	cleft lip and cleft palate; broad nasal bridge; bulbous nasal tip; deep-set eyes	bilateral epicanthal folds; upslanting palpebral fissures; bulbous nasal tip; enlarged columela; posteriorly rotated ears
Growth retardation	+		+	+	+		+	+
Intellectual disability	+	+	+	-	+	-	+	
Hearing loss	-	-	-	-			+	
Speech delay	+	+	+	+	+		+	+
Seizures	-	-	-	-	+	-	+	+
Short stature	-	-	-	-	+	-	+	
Poor attention	+	-	+	-	+	-	+	+
Heart defect	-	-	+	-		-	-	-
Limbs	-	Hypotonia	-	-	oligodactyly	-	-	Finger hyperconvex
Urogenital anomalies	-	-	-	+	+	+	-	+
Patient reported	Sehested et al. (2010) 1#	Sehested et al. (2010) 2#	Petrin et al. (2010)	Caselli et al. (2008)	Rossi et al. (2008)	Bisgaard et al. (2006)	Bisgaard et al. (2006)	Verma et al. (1992)	Fagan et al. (1994)
Sex	F	F	M	F	F	F	F	F	F
Age	42 years	34 years	-	-	-	-	-		
Cytogenetic location	7q34-q36.2	7q34-q36.2	7q33-q35	7q36.1-q36.2	7q33-q36.1	7q34-q36.2	7q34-q36.2	7q36.1-q36.2	7q35
Size of deletion	12.2 Mb	12.2 Mb	10 Mb	5.27 Mb	12 Mb	12.4 Mb	12.2 Mb	5.27 Mb	-
Brain structure abnormalies	-	-	cerebellar atrophy	hypoplasia of the corpus callosum	-	-	-	-	-
Facial features	hypertelorism; deep-set eyes; narrow palpebral fissures; bulbous nasal tip; broad nasal bridge; broad mouth; low-set ears	hypertelorism, deep-set eyes, narrow palpebral fissures, bulbous nasal tip, broad nasal bridge, broad mouth, and thick vermilion	broad nasal root	prominent forehead; deep set eyes; posteriorly angulated ears; bilateral epicanthal folds; flat nasal bridge; bulbous nasal tip; flat malar region	bulbous nasal tip; deep-set eyes; broad nasal bridge	round face; deep-set eyes; narrow palpebral fissures; low set ears; bulbous nasal tip; smooth philtrum; narrow upper lip	round face; deep-set eyes; narrow palpebral fissures; low set ears; bulbous nasal tip; smooth philtrum; narrow upper lip	cleft lip, cleft palate	bulbous nasal tip
Growth retardation	+	+	-	+	+	+	+	+	+
Intellectual disability	+	+	-	+	+	+	+	+	+
Hearing loss	-	-	-			+	+	+, Conductive	
Speech delay	+	+	+	+	+			+	+
Seizures	+	+	-	+	+	+	+	+, Febrile	-
Short stature	+	+	-	-	+	-	-		
Poor attention	+	+	-	-	-	+	+	-	-
Heart defect	-	-	-	-	-				
Limbs	-	-	broad halluces	Small hands	-	-	-	-	-
Urogenital anomalies	+	+	-		-	-	-	-	-

M, male; F, female.

In conclusion, we reported a *de novo* 9.4 Mb deletion ranging from 7q35 to q36.3 (chr7:147,493,985–156,774,460) in a patient with congenital brain dysplasia, developmental delay, and intellectual disability identified via SNP array analysis. Our study together with literature review indicated that 7q terminal deletion can be redefined as a contiguous 7q deletion syndrome, similar to other contiguous deletion syndromes, in which different regions and breakpoints gave an overlapping phenotype.

## Data Availability

The raw data supporting the conclusions of this article will be made available by the authors, without undue reservation.
