# Supplementary material for: Case Report: Congenital Brain Dysplasia, Developmental Delay and Intellectual Disability in a Patient With a 7q35-7q36.3 Deletion
Source: Front Genet. 2021 Dec 1;12:761003. doi: 10.3389/fgene.2021.761003 (PMC8671813; doi:10.3389/fgene.2021.761003)
Supplement: Supplementary file 1 [file DataSheet1.docx]

Figure S1


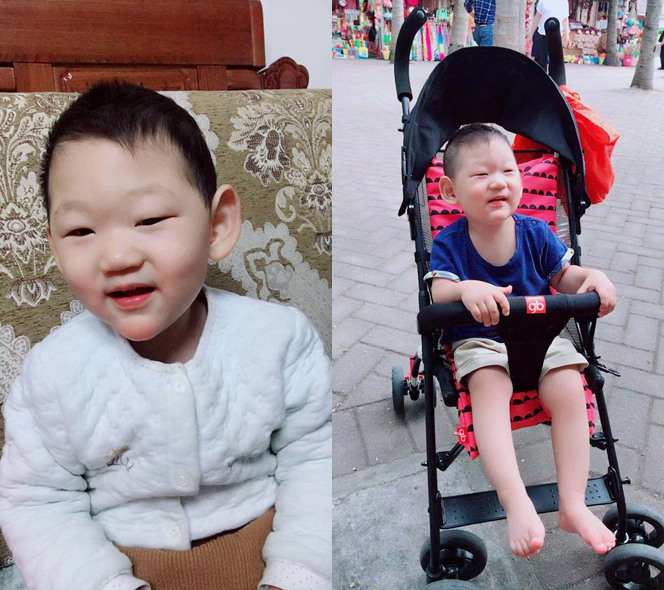


Figure S1. The photo of the proband.

Figure S2


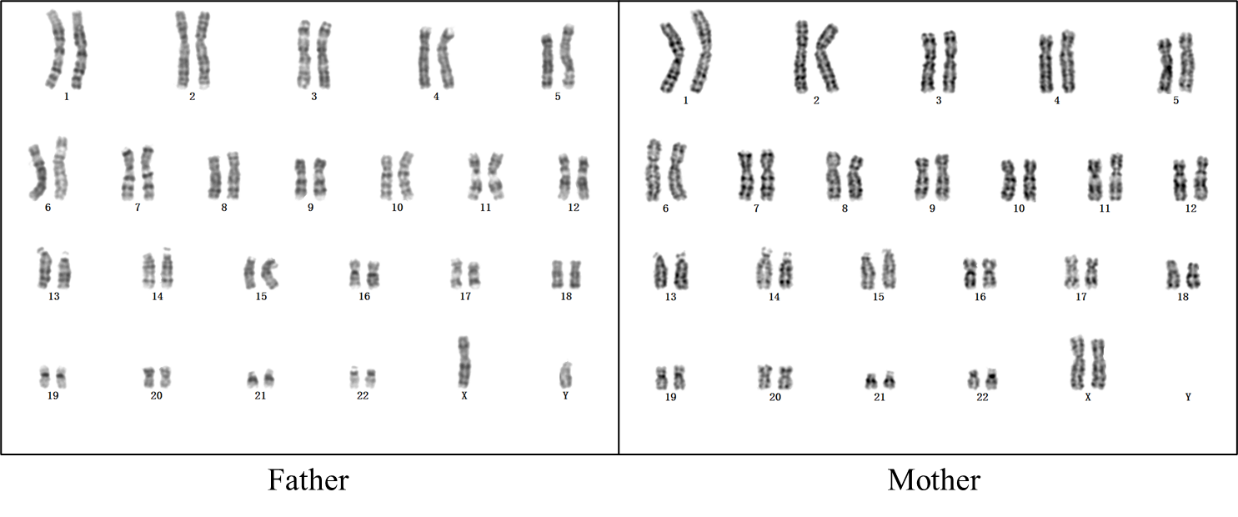


Figure S2. Banding cytogenetic result of patient.

Figure S3


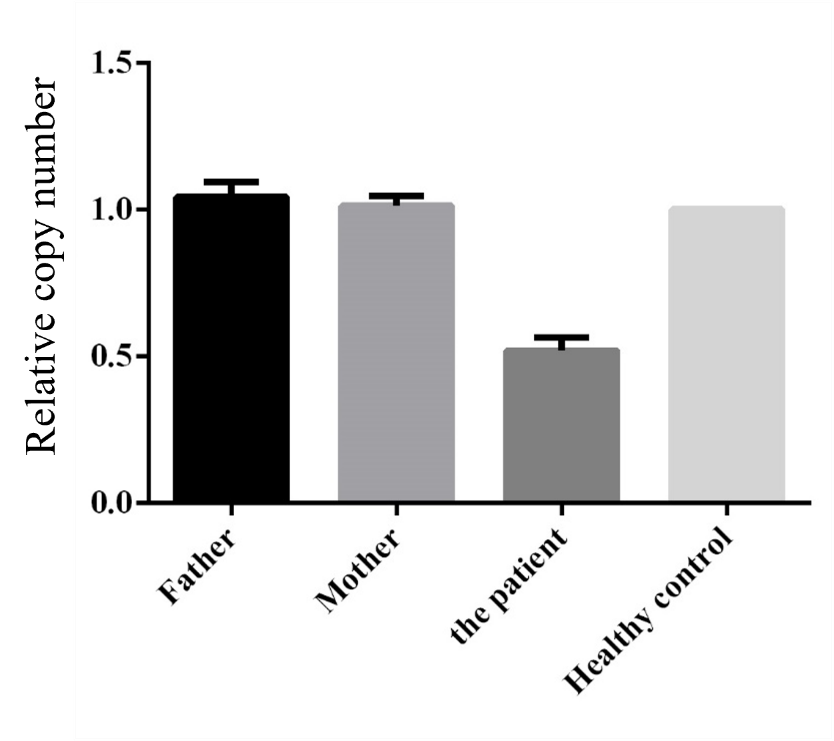


Figure S3. The real-time PCR data of the proband and his parent.
